# Renoprotective activity of sivelestat in severe acute pancreatitis in rats

**DOI:** 10.3892/etm.2013.1075

**Published:** 2013-04-24

**Authors:** HOUHONG WANG, A-MAO TANG, DAREN LIU, GUOGANG LI, LONGYUN YE, XIAOWEN LI, CHAO LI, LI CHEN

**Affiliations:** 1 Department of Surgery, Zhejiang University School of Medicine, Second Affiliated Hospital, Hangzhou, Zhejiang 310009;; 2 Zhejiang University of Traditional Chinese Medicine, Hangzhou, Zhejiang 310053, P.R. China

**Keywords:** acute pancreatitis, neutrophil elastase, sivelestat, renoprotection

## Abstract

Acute pancreatitis, affecting 382,014 individuals annually in China, is life-threatening in its severe form. Since acute pancreatitis-associated morbidity or mortality is attributable mainly to functional failure of the vital organs, significant research efforts have focused on the identification of novel agents with potential organ-protective properties in the hope of developing approaches to improve the outcome of acute pancreatitis. In a previous study, we demonstrated that sivelestat, a specific inhibitor of neutrophil elastase (NE), is effective in protecting against lung failure in rats with taurocholate-induced acute pancreatitis. As part of the analyses extended from that study, the present study aimed to evaluate the role of sivelestat in the protection against acute pancreatitis-associated renal injury. Renal histopathology and major renal function parameters were analyzed in renal tissue and blood specimens collected from rats with acute pancreatitis induced by the surgical administration of sodium taurocholate in the presence or absence of sivelestat treatment and in sham-operated control rats at various time-points. The extended analyses demonstrated that: i) sodium taurocholate induced apparent renal injury and dysfunction manifested by histological anomalies, including vacuolization and apoptosis of the cells of the tubular epithelial lining in the kidney, as well as biochemical aberrations in the blood (increases in levels of blood urea nitrogen, creatinine and tumor necrosis factor-α) and renal tissue (robust increases in NE activity and induced neutrophil chemoattractant-1 levels); and ii) sivelestat treatment effectively attenuated all taurocholate-induced histological anomalies and biochemical aberrations. These observations strongly suggest that the NE inhibitor, sivelestat, is effective in protecting against acute pancreatitis-associated renal injury.

## Introduction

Acute pancreatitis is a condition where inflammation occurs suddenly in the pancreas. The pancreas, located behind the stomach in the upper abdomen, produces digestive enzymes and the sugar-processing hormones, insulin and glucagon. Although the exact etiology of acute pancreatitis remains controversial (1), gallstones and heavy alcohol consumption are the two most common causes (2). With symptoms including a sudden onset of dull and steady pain in the upper abdomen, acute pancreatitis occurs at an incidence rate of 2.9 per 10,000 persons and affects 382,014 (0.029%) individuals annually in China (3).

Acute pancreatitis is mild in 80% of cases and severe in the remaining 20% of cases (2). Mild acute pancreatitis, also called edematous or interstitial pancreatitis, is defined as pancreatic inflammation and edema associated with minimal organ dysfunction, whereas severe acute pancreatitis is defined as pancreatic necrosis associated with secondary injury to extrapancreatic organs leading to multiple organ dysfunction syndrome (MODS) and/or local complications (4).

Mild acute pancreatitis usually resolves within a few days with conservative management. However, severe acute pancreatitis may be life-threatening and requires management in an intensive care unit. Although extensive research and clinical efforts have been made in the management of acute pancreatitis during the past few decades (5), to date no effective cure is available (6) and the mortality from severe acute pancreatitis remains high (7). Therefore novel therapeutic strategies are required to improve the outcomes of patients with severe pancreatitis.

Given that MODS is the primary cause of morbidity and mortality associated with severe acute pancreatitis, novel therapeutic approaches aiming to prevent injury of the vital organs have become a subject of intensive investigation. In a previous study, we assessed the potential of sivelestat, a competitive inhibitor of human neutrophil elastase (NE) (8), in the protection against acute pancreatitis-associated lung injury in a rat model (9). As an extension of the analyses in our previous study, the present study aimed to evaluate the ability of sivelestat to protect against renal injury in acute pancreatitis in rats.

## Materials and methods

### Animals, experimental design and specimen collection

Since this study was an extension of a previous study from our group, the animals and their allocation, as well as the methods of pancreatitis induction and sivelestat treatment, were the same as described in our previous study (9). In summary, adult male Sprague-Dawley rats were randomized into the following groups: i) the experimental acute pancreatitis (EAP) group in which rats were induced to develop acute pancreatitis by the administration of sodium taurocholate through laparotomy under anesthesia; ii) the EAP plus sivelestat treatment group in which rats were injected with 2 mg/kg sivelestat through the superficial dorsal vein of the penis immediately following EAP induction; and iii) the control group in which a sham laparotomy was performed. At 6, 12 and 24 h after sivelestat or vehicle treatment, six rats from each group were sacrificed by depletion. Immediately before and after animal sacrifice, blood samples were collected through cardiac puncture and serum was prepared. Tissue blocks of the kidneys and pancreas were excised and fixed in 10% formalin or snap-frozen in liquid nitrogen The study protocol was approved by the Institutional Review Board of Zhejiang University (Hangzhou, China).

### Histological examination

Formalin-fixed kidney tissue blocks were dehydrated, embedded in paraffin, sectioned at 5 *μ*m and stained with hematoxylin and eosin (H&E). Abnormalities, including vacuolization of the tubular epithelial lining in the subcapsular region, patchy areas of hemorrhage in the interstitium and necrosis in the epithelial lining of the tubules towards the medullary region were assessed by two independent pathologists who were blind to the study design and the specimen identities.

### Renal function test

Serum levels of blood urea nitrogen (BUN) and creatinine (CR) were determined using standard laboratory methods.

### Measurement of serum tumor necrosis factor-α (TNF-α)

Serum levels of TNF-α were determined using an immunoassay kit (Biosource, Grand Island, NY, USA) following the manufacturer’s instructions.

### Measurement of NE activity and cytokine-induced neutrophil chemoattractant-1 (CINC-1) level in renal tissue

Tissue homogenate was prepared from frozen renal specimens using the method described for the homogenate preparation of lung tissue in our previous study (9). NE activity was determined spectrophotometrically using a chromogenic substrate. Levels of CINC-1 were measured with a sandwich enzyme-linked immunosorbent assay (ELISA) kit (R&D Systems, Minneapolis, MN, USA).

### Statistical analysis

Data are expressed as arithmetic means ± standard deviation (SD) and analyzed with one-way analysis of variance (ANOVA) and Bonferroni test. SPSS software was used for statistical analyses (SPSS Inc., Chicago, IL, USA). P<0.05 was considered to indicate a statistically significant difference.

## Results

### Renal histopathology

**Figure 1.**
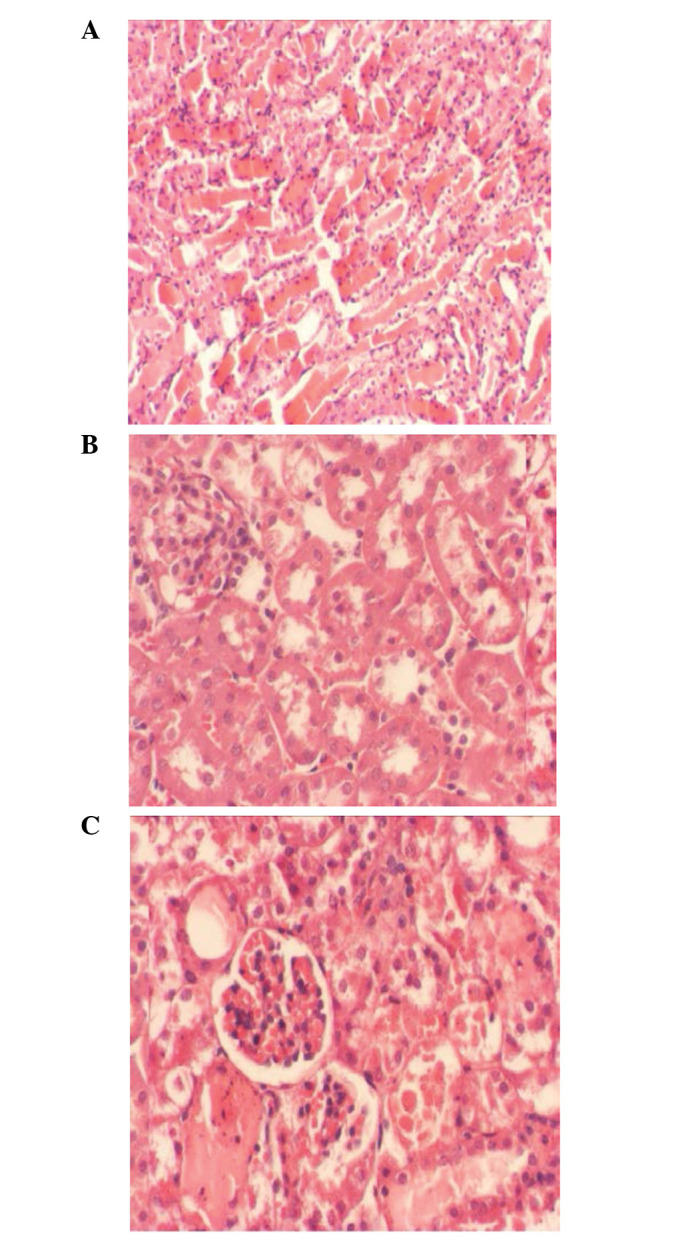
Images (magnification, ×200) of representative hematoxylin and eosin (H&E)-stained renal tissue sections at 24 h after acute pancreatitis induction. (A) Control; (B) experimental acute pancreatitis and (C) experimental acute pancreatitis plus sivelestat treatment.

Representative images of H&E-stained renal tissue sections 24 h after acute pancreatitis induction and sivelestat treatment are presented in Fig. 1. Structural anomalies were significant in the kidneys of rats treated with sodium taurocholate as compared with the control rats. Manifestations of these anomalies included apparent vacuolization of the tubular epithelial lining in the subcapsular region, patchy areas of hemorrhage in the interstitium and necrosis in the epithelial lining of the tubules towards the medullary region. Sivelestat treatment effectively ameliorated the sodium taurocholate-induced renal structure anomalies.

### Renal functions

As presented in Table I, the serum levels of BUN were consistent over time in the control rats; however, they were significantly increased at all three time-points in the rats with sodium taurocholate-induced acute pancreatitis (P<0.05). Sivelestat treatment effectively attenuated the taurocholate-induced increase in the serum levels of BUN. Similarly, serum levels of CR were significantly higher in the rats treated with sodium taurocholate than in the control rats at all three time-points (P<0.05) and sivelestat treatment returned the serum level of CR to the normal value observed in the control rats (Table II).

### Serum levels of TNF-α

The results of TNF-α measurement are summarized in Table III. The baseline level of TNF-α in the serum of control rats was between 4.17±1.04 and 5.73±0.81 pg/ml, with little difference between the three time-points (P<0.05). Sodium taurocholate induced a robust increase (P<0.001) in the serum level of TNF-α; however, this increase decreased in magnitude with time (22.8-fold at 6 h, 11.6-fold at 12 h and and 7.1-fold at 24 h). Sivelestat treatment significantly attenuated the taurocholate-induced increase in the serum level of TNF-α at all three time-points (P<0.01); however, it failed to return the level to normal.

### NE activity and CINC-1 concentration in renal tissue

As shown in Table IV, the NE activity in the renal tissue homogenate was consistent in control rats over time (P>0.05); however, it significantly (P<0.01) increased in animals with taurocholate-induced acute pancreatitis in a time-dependent manner (5.3-fold at 6 h, 8.2-fold at 12 h and 11.4-fold at 24 h). Although sivelestat treatment was not able to restore the normal baseline level of renal NE activity, it significantly attenuated the taurocholate-induced increase in NE activity at all time-points (P<0.01). Similar patterns of change were observed for the renal level of CINC-1 in the three groups; however, the magnitude of change induced by taurocholate was significantly larger (>100-fold) than that for the change in NE activity (Table V).

## Discussion

Acute pancreatitis may affect organs near to and distant from the pancreas, including the lungs, kidneys, liver and the cardiovascular and central nervous systems. According to the revised Atlanta classification of acute pancreatitis (10), organ failure is one of the major determinants of the severity of acute pancreatitis. While no organ failure is present in mild acute pancreatitis (the most common form of the disease) and organ failure is only transient in moderate acute pancreatitis, persistent (>48 h) and multiple organ failure or dysfunction (MODS) commonly occurs in severe acute pancreatitis (10). Moreover, previous clinical data has demonstrated that the number of organs experiencing function failure is positively associated with the mortality rate in severe acute pancreatitis (11). Therefore, protection against severe acute pancreatitis-associated organ failure improves the survival rate of patients with severe acute pancreatitis.

In a previous study (9), with the aim of identifying novel organ-protective agents, we evaluated the effects of the NE inhibitor, sivelestat, on lung dysfunction in a rat model of experimental acute pancreatitis. We observed the development of histopathological and biochemical abnormalities in the circulation, lungs and pancreas characteristic of acute pancreatitis in rats following the surgical administration of sodium taurocholate, as well as the effective attenuation of the taurocholate-induced abnormalities by sivelestat, suggesting a potential role for sivelestat in the protection against acute pancreatitis-associated pulmonary injury. Utilizing saved serum samples and renal tissue specimens from that study, in the present study we assessed the renoprotective activity of sivelestat.

We first assessed changes in the histology of kidneys from rats at 6, 12 and 24 h after taurocholate induction in the presence or absence of sivelestat treatment in a parallel comparison with sham-operated control animals. In agreement with results from a previous study (12), we observed histological anomalies in the renal tubules following taurocholate administration, confirming renal injury in rats with experimental acute pancreatitis. We also observed a significant amelioration in the taurocholate-induced renal histological changes in rats following sivelestat treatment, indicating that sivelestat has a beneficial effect on renal histology.

Kidney function tests are common laboratory tests used to evaluate how well the kidneys are working. To assess changes in renal function in the different groups, levels of BUN and CR were measured in the saved aliquots of serum samples collected in our previous study. Significant elevations were detected for BUN and CR in rats following surgery, compared with the corresponding baseline level in control animals. Sivelestat treatment significantly improved these renal function parameters. In the literature, to the best of our knowledge, there are no reports concerning the beneficial effects of sivelestat on BUN and CR, the major parameters of renal function. Kumasaka *et al* observed a beneficial effect of sivelestat on proteinuria in nephritis rats (13). Kumasaka’s observations and our own suggest a beneficial effect for sivelestat on renal function.

We also assessed changes in other renal function variables, including serum levels of TNF-α, NE activity and CINC-1 concentration in renal tissue. For the first time, we observed that sivelestat is able to significantly improve these variables.

## Figures and Tables

**Table I.** Serum levels of BUN (mmol/l) in the different groups at various time-points.

Group	6 h	12 h	24 h
Control	5.06±0.75	5.13±1.05	5.22±0.78
AP	16.82±1.51^a^	17.30±1.98^a^	19.62±2.04^a^
AP+S	11.57±1.92^b^	11.94±2.06^b^	12.43±2.15^b^

a α=0.05, between the acute pancreatitis (AP) and control groups at the same time-point.

b α=0.05 between the AP and AP plus sivelestat treatment (AP+S) groups and between the AP+S and control group at the same time-point. BUN, blood urea nitrogen.

**Table II.** Serum levels of creatinine (*μ*mol/l) in the different groups at various time-points.

Group	6 h	12 h	24 h
Control	21.06±1.75	21.73±1.51	22.03±1.69
AP	35.42±1.90^a^	36.81±1.84^a^	38.42±2.06^a^
AP+S	23.79±1.92^b^	24.54±1.07^b^	27.23±1.85^b^

a α=0.05 between the acute pancreatitis (AP) and control groups at the same time-point.

b α=0.05 between the AP and AP plus sivelestat treatment (AP+S) groups at the same time-point.

**Table III.** Serum levels of TNF-α (pg/ml) in the different groups at various time-points.

Group	6 h	12 h	24 h
Control	4.17±1.04	5.73±0.81	5.34±1.20
AP	95.12±21.42^a^	66.48±27.94^a^	38.12±22.17^a^
AP+S	63.77±25.92^b^	44.54±23.07^b^	26.23±19.85^b^

a α=0.05 between the acute pancreatitis (AP) and control groups at the same time-point.

b α=0.05 between the AP and AP plus sivelestat treatment (AP+S) groups at the same time-point. TNF, tumor necrosis factor.

**Table IV.** Neutrophil elastase activity (pg/ml) in the renal tissue in the different groups at various time-points.

Group	6 h	12 h	24 h
Control	1.35±0.37	1.42±0.28	1.34±0.25
AP	7.14±1.35^a^	11.65±1.98^a^	15.37±2.14^a^
AP+S	4.36±1.92^b^	6.89±2.07^b^	9.23±1.85^b^

a α=0.05 between the acute pancreatitis (AP) and control groups at the same time point.

b α=0.05 between the AP and the AP plus sivelestat treatment (AP+S) groups at the same time-point.

**Table V.** CINC-1 concentration (pg/g) in renal tissue in the different groups at various time-points.

Group	6 h	12 h	24 h
Control	52.23±3.77	57.42±5.34	61.34±7.85
AP	4500.14±538.30^a^	5374.65±577.48^a^	6208.37±534.23^a^
AP+S	3409.71±421.92^b^	4518.89±378.16^b^	5400.32±456.80^b^

a α=0.05 between the acute pancreatitis (AP) and control groups at the same time-point.

b α=0.05 between the AP and AP plus sivelestat treatment (AP+S) groups at the same time-point. CINC-1, cytokine-induced neutrophil chemoattractant-1.
